# Supplementary material for: NKB signaling in the posterodorsal medial amygdala stimulates gonadotropin release in a kisspeptin-independent manner in female mice
Source: eLife. 2018 Dec 19;7:e40476. doi: 10.7554/eLife.40476 (PMC6300354; doi:10.7554/eLife.40476)
Supplement: Transparent reporting form [file elife-40476-transrepform.docx]

***eLife’s* transparent reporting form**

We encourage authors to provide detailed information *within their submission* to facilitate the interpretation and replication of experiments. Authors can upload supporting documentation to indicate the use of appropriate reporting guidelines for health-related research (see [EQUATOR Network](http://www.equator-network.org/%20)), life science research (see the [BioSharing Information Resource](https://biosharing.org/)), or the [ARRIVE guidelines](http://www.plosbiology.org/article/info:doi/10.1371/journal.pbio.1000412) for reporting work involving animal research. Where applicable, authors should refer to any relevant reporting standards documents in this form.

If you have any questions, please consult our Journal Policies and/or contact us: [editorial@elifesciences.org](mailto:editorial@elifesciences.org).

**Sample-size estimation**

- You should state whether an appropriate sample size was computed when the study was being designed
- You should state the statistical method of sample size computation and any required assumptions
- If no explicit power analysis was used, you should describe how you decided what sample (replicate) size (number) to use

Please outline where this information can be found within the submission (e.g., sections or figure legends), or explain why this information doesn’t apply to your submission:

Power calculation was performed using Wilcoxon rank-sum method assuming non-normal distribution of the outcome with the help of the Harvard Catalyst Biostatistics Core. A sample size of 10 animals in each group will allow us to detect a difference between two groups with a two-sided alpha of 0.05 and a power of at least 84%. The sample size calculation accounts for 12.5% loss due to animals that did not receive the proper dose of the virus because of misplacement of the injection site or adverse reaction to the injection. These possible non-responders will be determined post mortem as described in the proposal. The calculation was performed using nQuery Adviser® v 7.0. In experiments where two groups are compared, student *t*-test will be performed. For comparisons of more than two groups, the statistical level will be determined using ANOVA followed by the corresponding *post hoc* test depending on what kind of ANOVA (One Way or Two Way) is used.

**Replicates**

- You should report how often each experiment was performed
- You should include a definition of biological versus technical replication
- The data obtained should be provided and sufficient information should be provided to indicate the number of independent biological and/or technical replicates
- If you encountered any outliers, you should describe how these were handled
- Criteria for exclusion/inclusion of data should be clearly stated
- High-throughput sequence data should be uploaded before submission, with a private link for reviewers provided (these are available from both GEO and ArrayExpress)

Please outline where this information can be found within the submission (e.g., sections or figure legends), or explain why this information doesn’t apply to your submission:

This information is included for each experiment in the methods and the specific statistical analysis indicated in the figure legend. Animals without proper stereotactic injection in the targeted brain area (assessed post-mortem) were excluded from the experiment.

**Statistical reporting**

- Statistical analysis methods should be described and justified
- Raw data should be presented in figures whenever informative to do so (typically when N per group is less than 10)
- For each experiment, you should identify the statistical tests used, exact values of N, definitions of center, methods of multiple test correction, and dispersion and precision measures (e.g., mean, median, SD, SEM, confidence intervals; and, for the major substantive results, a measure of effect size (e.g., Pearson's r, Cohen's d)
- Report exact p-values wherever possible alongside the summary statistics and 95% confidence intervals. These should be reported for all key questions and not only when the p-value is less than 0.05.

Please outline where this information can be found within the submission (e.g., sections or figure legends), or explain why this information doesn’t apply to your submission:

Description of the statistical analysis was included in the methods and figure legends.

(For large datasets, or papers with a very large number of statistical tests, you may upload a single table file with tests, Ns, etc., with reference to sections in the manuscript.)

**Group allocation**

- Indicate how samples were allocated into experimental groups (in the case of clinical studies, please specify allocation to treatment method); if randomization was used, please also state if restricted randomization was applied
- Indicate if masking was used during group allocation, data collection and/or data analysis

Please outline where this information can be found within the submission (e.g., sections or figure legends), or explain why this information doesn’t apply to your submission:

Animals were randomized into each experimental group. All samples for analysis are coded such that phenotyping and IHC are done blind to treatments. The code is only broken at the end of an experiment to allow assignment of final data to groups. For *in vivo* experiments that are performed on different days, animals from different groups will be mixed so any potential variation in the response of the animals due to time is normalized across all groups.

**Additional data files (“source data”)**

- We encourage you to upload relevant additional data files, such as numerical data that are represented as a graph in a figure, or as a summary table
- Where provided, these should be in the most useful format, and they can be uploaded as “Source data” files linked to a main figure or table
- Include model definition files including the full list of parameters used
- Include code used for data analysis (e.g., R, MatLab)
- Avoid stating that data files are “available upon request”

Please indicate the figures or tables for which source data files have been provided:

Figures of numerical data were created and analyzed using Graph Pad Prism.
